# Case Report: Two cases of IgG4-related kidney disease with monoclonal gammopathy

**DOI:** 10.3389/fimmu.2025.1539441

**Published:** 2025-06-24

**Authors:** Qian Dong, Xuanli Tang, Jiang Liu, Pengjie Xu

**Affiliations:** ^1^ The Nephrology Department, Lihuili Hospital Affiliated with Ningbo University, Ningbo, China; ^2^ The Nephrology Department, Hangzhou Traditional Chinese Medicine Hospital, Hangzhou, China

**Keywords:** IgG4-related kidney disease, IgG4-related tubulointerstitial nephritis (IgG4-TIN), secondary membranous nephropathy (SMN), monoclonal gammopathy of renal significance (MGRS), monoclonal gammopathy of undetermined significance (MGUS)

## Abstract

**Introduction:**

IgG4-related disease (IgG4-RD) is a newly classified but poorly understood immune-medicated systemic disease. Monoclonal gammopathy encompasses a spectrum of disorders driven by clonal plasma cell proliferation, leading to the presence of monoclonal immunoglobulins (M-proteins) in serum or urine. IgG4-related nephropathy with associated monoclonal gammopathy is rare, and its etiology is poorly understood.

**Case presentation:**

This article reports two rare cases of IgG4-related kidney disease combined with monoclonal gammopathy. Case 1 was a 69-year-old male presented with renal insufficiency and mild proteinuria, along with significant elevations of IgG, IgG4, eosinophils, and IgE, and severe reductions in complement C3 and C4. Serum free light chains κ and λ levels were elevated, monoclonal IgG-λ protein was detected, and lymphadenopathy was observed. Renal biopsy revealed multi-focal and flaky storiform fibrosis. In the renal interstitium, IgG4-positive cells >10/high-power field (HPF), IgG4/IgG >40%. Interstitial inflammatory cells κ were scattered weakly positive, and λ was diffuse strong positive. Bone marrow aspiration did not show obvious plasmablast or abnormal plasma cell clones, but eosinophils were significantly elevated. Case 2 was a 71-year-old male who presented with renal insufficiency and massive proteinuria, along with elevated IgG and IgG4 levels. Serum free light chain κ and λ were elevated, and monoclonal IgM-κ was identified. PLA2R testing was negative. Renal biopsy revealed secondary membranous nephropathy (SMN) without evidence of monoclonal renal injury. Bone marrow aspiration showed evident eosinophils, mature plasma cells accounted for 3.5% and no express light chain restriction.

**Conclusions:**

Case 1 was diagnosed with IgG4-TIN combined with MGRS, which showed significant renal and hematological improvement after corticosteroid therapy. Case 2 was diagnosed as secondary membranous nephropathy combined with MGUS, but IgG4-MN cannot be ruled out. His renal function improved with methylprednisolone and rituximab treatment, but the M-protein persisted. We review the related diagnosis and treatment to provide more information for clinical practice.

## Introduction

IgG4-related disease (IgG4-RD) is a newly defined, immune-mediated chronic inflammatory disorder characterized by fibrosis. Monoclonal gammopathy comprises a group of disorders characterized by clonal plasma cell expansion, which produces monoclonal immunoglobulins (M-proteins) detectable in serum or urine. IgG4-related kidney disease concurrent with monoclonal gammopathy is extremely rare. Here, we report two cases of this condition and review the relevant literature.

## Case presentation

### Case 1

A 69-year-old Chinese male was admitted to hospital for investigation of renal dysfunction on October 17, 2023. Five months prior, he experienced increased nocturnal urination accompanied by foamy urine, hematuria, blurred vision, fatigue, and poor appetite, without joint pain or lower extremity edema. His laboratory results showed serum creatinine of 206.8 µmol/L and a urine protein-to-creatinine ratio (UPCR) of 1.39 mg/mg. He was diagnosed with hypertension for over six months and anemia for four years. No abnormalities found on physical examination. The clinical findings on admission are summarized in **Table 1**. In brief, the eosinophil count was markedly increased (300%). The IgG and IgG4 levels were markedly increased (31.50 and 31.53 g/L, respectively). Complement 3 (C3) and Complement 4 (C4) were significantly decreased (38.90 and 1.67 mg/dl, respectively). Free light chain (FLC) κ 1021.98 mg/L, FLC λ 204.22 mg/L, κ/λ 5.00, monoclonal IgG-λ protein present. Bilateral inguinal lymph nodes were enlarged. Renal ultrasound showed normal size and morphology of both kidneys, clear corticomedullary demarcation. We further performed a bone marrow aspiration biopsy on him, which revealed that a moderately low amount of nucleated cells, 18.5% lymphocytes, 5.65% eosinophils, and no obvious primary and juvenile plasma cells.

**Table 1:** Clinical findings of the patients on admission.

	Case 1	Case 2	Normal Range
Urinalysis			
Urinary protein	(+-)	(2+)	(-)
24h UTP (mg/d)	Not tested	4858.60	10-140
UPCR (mg/mg)	1.39	2.08	0.00-0.02
Blood Chemistry			
Hb (g/L)	99	110	130-175
EOS(*10^9^/L)	1.58	0.26	0.02-0.52
CRP (mg/L)	3.50	3.50	0.00-10.00
ALB (g/L)	27.50	32.30	40.00-55.00
Scr (μmol/L)	217.10	225.10	57.00-111.00
eGFR (mL/min/1.73m²)	25.80	25.40	90.00-120.00
Uric acid (μmol/L)	368	668.00	208-428
Immuno-serological findings			
Ig A (g/L)	1.61	3.76	0.82-4.53
Ig G (g/L)	31.50	44.70	7.51-15.60
Ig M (g/L)	1.19	5.72	0.46-3.04
IgG4 (g/L)	31.53	3.32	<2.00
Ig E (IU/mL)	711.00	1220	0.00-165.00
C3 (mg/dL)	38.90	72.30	79.00-152.00
C4 (mg/dL)	1.67	14.40	16.00-38.00
FLC κ (mg/L)	1021.98	287.59	3.30-19.40
FLC λ (mg/L)	204.22	189.40	5.71-26.30
FLC κ/λ ratio	5.00	1.52	0.26-1.65
Immunofixation electrophoresis	IgG-λ	IgM-λ	Not detected
Serum protein electrophoresis	M-protein	M-protein	Not detected
Anti-nuclear antibodies	1:100	1:320	(-)
Anti-double-stranded DNA	(-)	(-)	(-)
Anti-Sm antibodies	(-)	(-)	(-)
Inguinal lymph nodes	Enlarged	Normal	
Renal ultrasound	Normal	Normal	
Bone marrow biopsy	No primary and juvenile plasma cells	no express light chain restriction	

UPCR, Urine Protein-to-Creatinine Ratio; 24h UTP, 24-h urinary total protein; Hb, Hemoglobin; EOS, Eosinophil; CRP, C-reactive protein; ALB, Albumin; Scr, Serum Creatinine; eGFR, Estimated Glomerular Filtration Rate; Ig, immunoglobulin; C, Complement; FLC, Free light chain.

**Figure 1:**
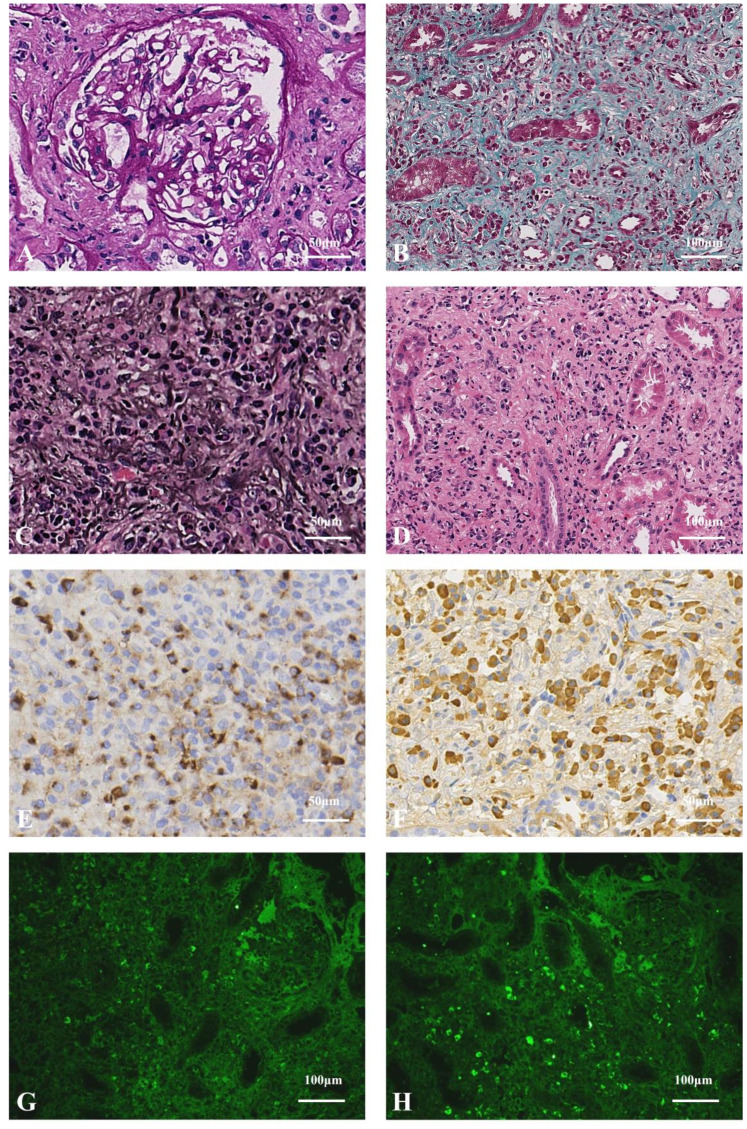
**(A)** Mild glomerular lesions (PAS staining×400). **(B)** Collagen-rich, solitary fibrosis (Masson staining×200). **(C)** Storiform fibrosis and bird's eye sign changes, which is specific morphological features in IgG4-TIN (PASM staining×400). **(D)** Massive renal interstitial inflammatory cells (mainly plasma cells, accompanied by lymphocytes, monocytes, and eosinophils) infiltration (HE staining×200). **(E)** The number of IgG4-positive cells>50/HPF (immunohistochemistry×400). **(F)** IgG-positive cells, IgG4/IgG>40% (immunohistochemistry×400). **(G)** Sparse, weakly positive κ light chains (paraffin fluorescence×200). **(H)** Diffuse, strongly positive λ light chains, which revealed monoclonal lymphoplasma infiltration (paraffin fluorescence×200).

In order to make a definitive diagnosis, he underwent routine ultrasonic guided right kidney biopsy. By light microscopy, six minimal glomerular changes with focal, interstitial areas exhibited isolated, collagen-rich severe fibrosis (about 40% area) (**Figures 1A, B**), with bird’s eye and storiform fibrosis patterns (**Figure 1C**), and diffuse infiltration of inflammatory cells were noted (predominantly plasma cells, **Figure 1D**). Immunofluorescence showed IgG and its subtypes, IgA, C3, and C1q were all negative, and IgM (+/-). CD38 and IgG4 were diffusely positive in the renal interstitium; the strong positivity for IgG4 in the renal interstitium with more than 50 cells per high power field, and IgG4/IgG ratio >40% (**Figures 1E, F**). Paraffin fluorescence: κ light chain was weakly positive in a small amount (average 10 cells/HPF), and λ light chain was diffusely strong positive (average 100 cells/HPF), and the distribution of the two was significantly different (*P <*0.05) (**Figures 1G, H**). Electron microscopy: no significant glomerular basement membrane lesions, with foot process effacement. We consider he has IgG4-related tubulointerstitial nephritis (IgG4-TIN) with monoclonal gammopathy of renal significance (MGRS).

**Figure 2:**
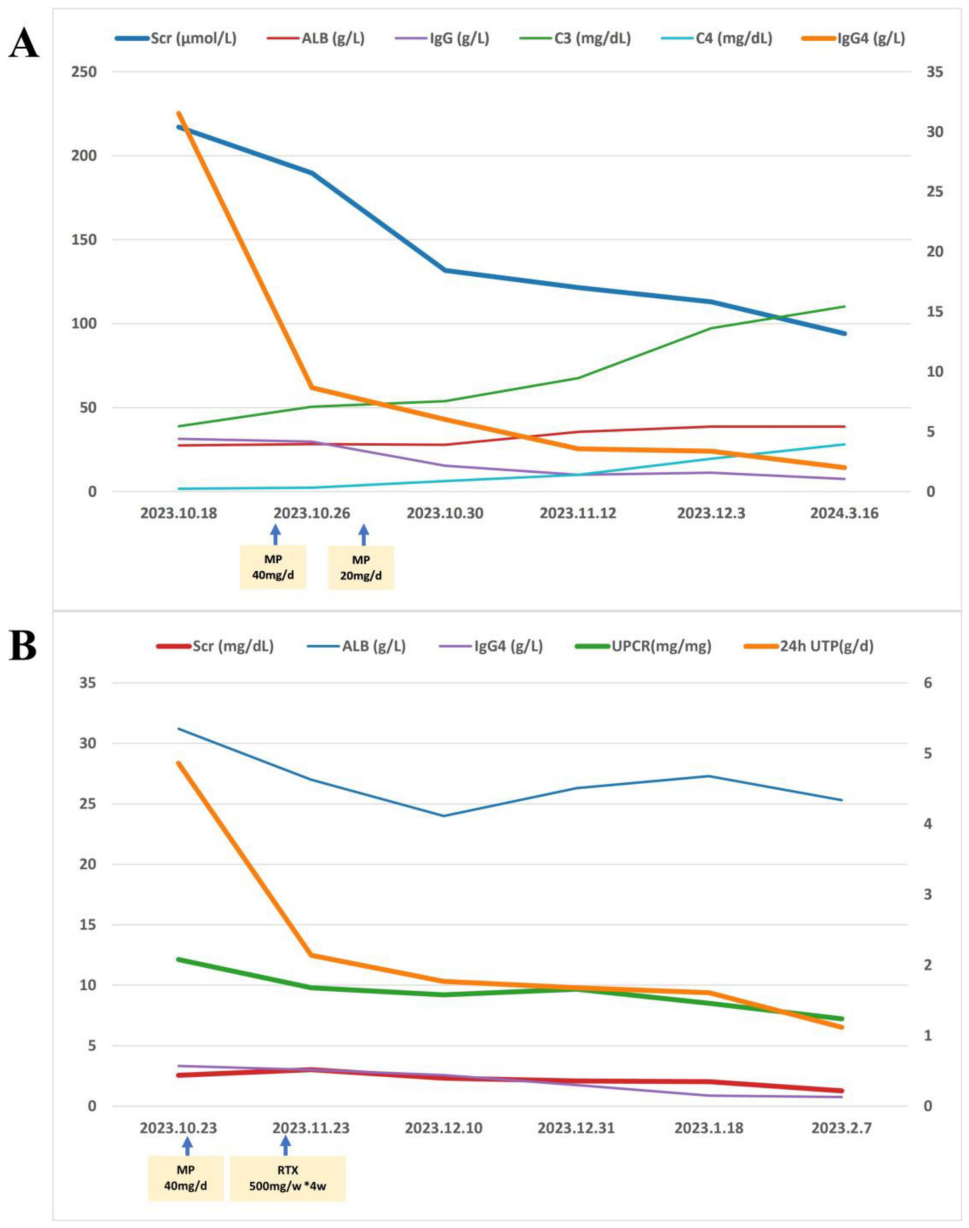
Treatment outcomes in two cases of IgG4-related nephropathy. **(A)** Case 1 received intravenous methylprednisolone (MP; 40 mg/day for 3 days) followed by oral MP (20 mg/day, tapered). At 20-week follow-up, improvements were observed, including decreased serum creatinine (Scr), IgG and IgG4 levels, alongside increased albumin (ALB), complement C3 and C4. **(B)** Case 2 received intravenous MP (40 mg/day for 3 days) followed by oral MP (24 mg/day, tapered) combined with rituximab (500 mg weekly ×4). By 14-week follow-up, reductions were seen in Scr, 24-hour urinary total protein (24hUTP), urine protein-to-creatinine ratio (UPCR) and IgG4, with stable ALB levels.

The patient was prescribed methylprednisolone (MP) at an initiated dose of 40 mg/d ivgtt for 3 days, followed by 20 mg orally then tapered. Symptoms relieved soon. Serum creatinine declined gradually to 94.10 μmol/L and albumin rose to 38.60 g/L at the 20 weeks after MP-one initiation. The IgG and IgG4 levels were markedly decreased (7.51and 2.00 g/L, respectively). C3 and C4 levels were significantly increased (110.20 and 28.00 mg/dl, respectively). Regrettably, due to the limitations of medical insurance and the patient’s financial situation, we were unable to recheck the free light chain levels after treatment (**Figure 2A**). No monoclonal protein was detected. Bilateral inguinal lymph nodes returned to normal.

### Case 2

A 71-year-old Chinese male was admitted to hospital for investigation of hypergammaglobulinemia on October 23, 2023. One month ago, the patient developed bilateral lower limb edema accompanied by foamy urine, without rash, abdominal pain, or diarrhea. Examination showed abnormal increase of monoclonal IgM-κ protein. He had thrombotic thrombocytopenic purpura treated successfully with rituximab, corticosteroids and intravenous immunoglobulin six years ago. No abnormalities found on physical examination. The clinical findings on admission are summarized in **Table 1**. In brief, Serum creatinine 225.10 µmol/L, 24-hour urine protein 4858.60 mg/d, UPCR 2.08 mg/mg. The IgG and IgG4 levels were significantly increased (44.70 and 3.32 g/L, respectively). FLC κ 287.59 mg/L, FLC λ 189.40 mg/L, κ/λ 1.52, monoclonal IgM-λ protein present. Phospholipase A2 Receptor (PLA2R) was negative. Pelvic X-ray revealed bilateral degenerative changes in the hip joints, possible sacroiliitis on both sides. Renal ultrasound showed normal size and morphology of both kidneys, enhanced cortical echogenicity, and poorly defined corticomedullary demarcation. Bone marrow aspiration showed that eosinophils are easily visible in the marrow smear, mature plasma cells account for 3.5%, and no express light chain restriction.

**Figure 3:**
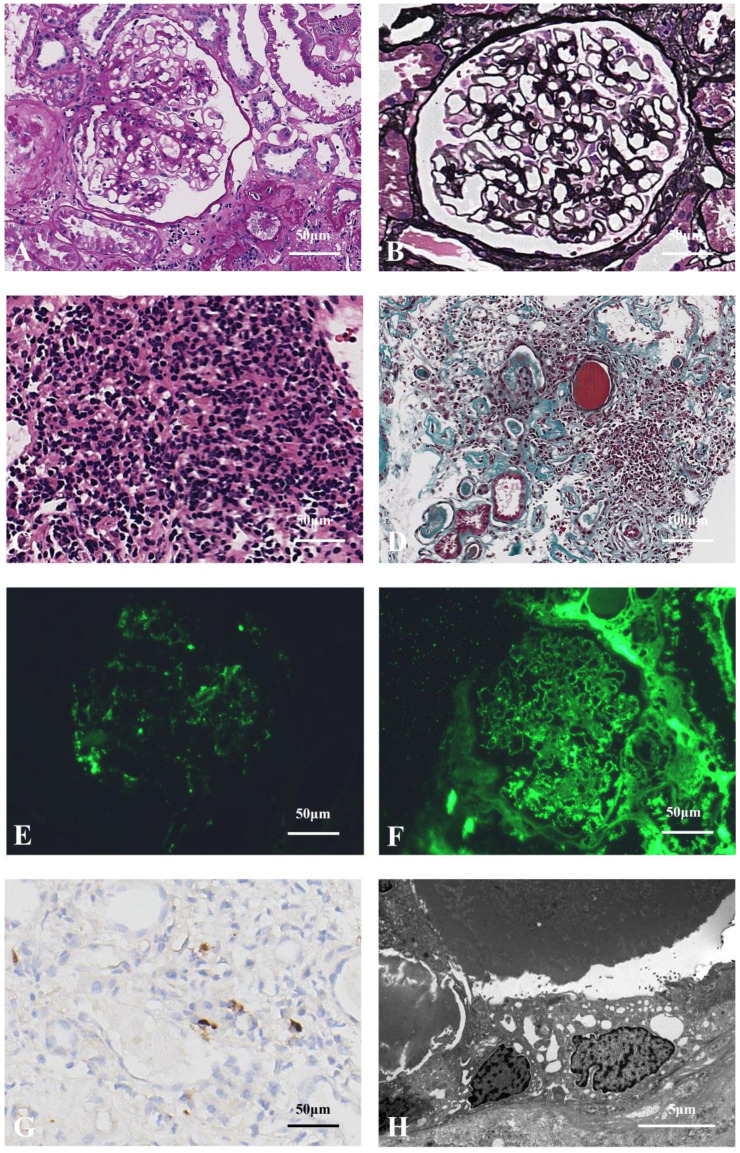
**(A)** Enlarged glomerular with thickened basement membrane (PAS staining×400). **(B)** Thickened glomerular basement membrane with slight vacuolar degeneration, which shows the specific morphological feature of MN (PASM staining×400). **(C)** Multifocal inflammatory cell (mainly lymphocyte) infiltration in the renal interstitium (HE staining×400). **(D)** Infiltration of inflammatory cells in the renal interstitium with fibrosis and tubular atrophy, without the storiform feature (Masson staining×200). **(E)** Glomerular IgG4 granular positive distribution along vascular loops (paraffin fluorescence×400). **(F)** Glomerular IgG granular positive distribution along the vascular loops (paraffin fluorescence×400). **(G)** Scattered IgG4-positive cells (<10/HP) observed in the renal interstitium, which did not meet the diagnostic criteria for IgG4-TIN (immunohistochemistry×400). **(H)** No electron-dense deposits identified in the renal tubular basement membrane (electron microscopy×6000).

The patient underwent routine ultrasonic guided right kidney biopsy. By light microscopy, 22 glomeruli with 13 showing global sclerosis, 1 segmental sclerosis, and 1 large fibroblastic crescent. The remaining glomeruli were enlarged with diffuse thickening of the basement membrane with subepithelial fuchsinophilic protein deposition. PASM staining revealed vacuolar degeneration (**Figures 3A, B**). Multifocal inflammatory cell infiltration with fibrosis in the renal interstitium was observed, with no obvious bird’s eye sign or storiform fibrosis (**Figures 3C, D**). Immunofluorescence: IgG (++), IgG4 (+), IgA (+), IgM (+), C3 (-), C1q (+), κ (+), λ (+) were positively deposited along the capillary loops, and the other IgG subtypes, PLA2R, and THSD7A were all negative (**Figures 3E–G**). Paraffin fluorescence: There was no significant difference in the positive κ and λ in the glomeruli and interstitium. No glomeruli were observed under electron microscopy, and no electron-dense deposits were found in the tubular basement membrane (**Figure 3H**). We consider he has secondary membranous nephropathy (SMN) combined with monoclonal gammopathy of undetermined significance (MGUS), but IgG4-MN cannot be ruled out.

The patient was prescribed MP at an initiated dose of 40 mg/d for 3 days, followed by 24 mg orally then tapered. With combination of rituximab (RTX, 500 mg weekly for four weeks). The serum creatinine decreased to 1.27 mg/dL, and 24-hour urine protein decreased to 1.12 g/d at the 14 weeks after treatment, IgG4 levels returned to normal range. Albumin did not rise significantly (**Figure 2B**). Regrettably, due to the limitations of medical insurance and the patient’s financial situation, we were unable to recheck the free light chain levels after treatment. However, the M-protein remained of the IgM-λ type.

## Discussion

IgG4-related disease (IgG4-RD) is a newly defined immune-mediated chronic inflammatory disease accompanied by fibrosis. The primary pathological manifestations include lymphoplasmacytic infiltration dominated by IgG4-positive plasma cells, storiform fibrosis, occlusive phlebitis, and eosinophil infiltration (1). There is limited published data on the global incidence and prevalence of IgG4-RD. Most patients are male, over 50 years old (2), and respond to glucocorticoid therapy within weeks. The pathogenesis of IgG4-RD is complex, with recent studies identifying a significant role for CD4+ cytotoxic T lymphocytes (CTLs) (3, 4). In addition, genetic risk and bacterial infection are also implicated in the pathogenesis of IgG4-RD (5, 6). In IgG4-related disease (IgG4-RD), kidney involvement is termed IgG4-related kidney disease (IgG4-RKD). The most common manifestation is tubulointerstitial nephritis (TIN), while membranous nephropathy (MN) represents the predominant glomerular lesion, occurring in approximately 7% of cases. MN often coexists with TIN, but can also present independently (7, 8). Saeki T et al. (2020) developed comprehensive diagnostic criteria for IgG4-RKD, encompassing renal functional, serological, radiological, and histopathological parameters to standardize clinical diagnosis (9). The key diagnostic criteria of IgG4-RKD include: (1) renal manifestations (e.g., acute or chronic renal dysfunction, proteinuria, hypocomplementemia) and imaging findings (e.g., bilateral hypoechoic masses, diffuse renal enlargement, or pelvicalyceal wall thickening); (2) elevated serum IgG4 levels (typically >1.35 g/L); (3) histopathological evidence of IgG4-positive plasma cell infiltration (>10 cells/HPF) and characteristic fibrosis (storiform fibrosis or obliterative phlebitis); (4) concurrent IgG4-related diseases (e.g., autoimmune pancreatitis, dacryoadenitis) (9, 10). In Case 1, the patient presented with renal insufficiency, mild proteinuria, elevated IgG4 levels, and lymphadenopathy. Renal biopsy showed multifocal and flaky renal interstitial fibrosis, with >10 IgG4-positive cells/HPF and IgG4/IgG >40%. Based on the diagnostic criteria, IgG4-TIN can be confirmed. In case 2, since the patient does not exhibit systemic manifestations of IgG4 and has weak IgG4 deposition in renal tissue, it does not meet the diagnostic criteria for IgG4-related kidney disease (IgG4-RKD).

The clinical manifestations of IgG4 are highly variable. They typically include organ or tissue swelling and sclerosis, along with elevated serum IgG4 levels. Diagnosis requires differentiation from inflammatory, lymphomatous, or malignant conditions (10–12). We reviewed the literature and found that there are several reports of cases where elevated serum IgG4 levels were ultimately diagnosed as lymphoma. Ohta et al. reported an elderly patient with elevated blood IgG4 levels and bilateral swelling of the parotid and submandibular glands, who was eventually diagnosed with marginal lymphoma. After rituximab treatment, the patient’s submandibular gland swelling improved, but the blood IgG4 level remained high (13). Wu et al. reported three cases of IgG4-related eye disease that eventually progressed to ocular adnexal marginal zone B-cell lymphoma (14). Similarly, Nishida et al. described a case in which the patient was initially treated with corticosteroids but diagnosed with marginal zone B-cell lymphoma of the ocular adnexa four years later (15). Hui W et al. recently reported a case of IgG4-TIN combined with interstitial inflammatory cell λ light chain restriction, which was eventually diagnosed as lymphoma. They speculated that the patient’s diffuse large B-cell lymphoma was transformed from a pre-existing but possibly underdiagnosed extranodal marginal zone lymphoma (16). In our Case 1, although monoclonal plasma cell infiltration was observed in the renal interstitium, it remains unclear whether this was related to lymphadenopathy, as no lymph node biopsy was performed. However, follow-up results showed a significant decline in IgG4 levels, normalization of lymph node size, and improvement in renal function after corticosteroid therapy, suggesting a possible link between IgG4-RKD and M-protein. Long-term prognosis, especially hematological changes, requires close monitoring. In Case 2, the renal biopsy findings—notably enlarged glomeruli with diffuse basement membrane thickening—favored a diagnosis of secondary membranous nephropathy (SMN), given the absence of systemic IgG4-related disease manifestations, weak IgG4 deposition in renal tissue, and negative PLA2R staining. Nonetheless, IgG4-related membranous nephropathy (IgG4-MN) remains a possible differential diagnosis.

Monoclonal gammopathy is a clonal disorder of plasma cells or B-lymphocytes, characterized by the production of a M-protein detectable in serum or urine, and encompassing a spectrum from asymptomatic conditions (e.g., MGUS) to malignancies (e.g., multiple myeloma). Based on its clinical significance and etiology, monoclonal gammopathy can be categorized into the following types: monoclonal gammopathy of undetermined significance (MGUS) and monoclonal gammopathy of clinical significance (MGCS). MGUS usually causes no symptoms and may be discovered incidentally when tests for other diseases are performed. Its diagnostic criteria include: A serum monoclonal protein level of less than 30 g/L. A percentage of plasma cells in the bone marrow of less than 10%. Absence of clinical manifestations associated with M protein such as anemia, hypercalcemia, osteolytic lesions, or renal insufficiency (17, 18). The prevalence of MGUS in the Chinese population is relatively high, particularly in the elderly, with an incidence of approximately 1.11% in individuals over 50 years old and increasing to 2.57% in those over 70. Rates are slightly higher in Western countries, but the difference is not significant (19). The pathogenesis of MGUS remains unclear but is generally thought to involve genetic alterations (e.g., IgH translocation, chromosome 13 deletion), angiogenesis, cytokines, myeloma bone disease, and Helicobacter pylori (17). MGUS generally requires no treatment but warrants ongoing monitoring given its risk of transformation to malignant plasma cell disorders. If patients meet the diagnostic criteria for MGUS and exhibit kidney damage caused by M-protein, they should be diagnosed with monoclonal gammopathy of renal significance (MGRS) (20). In our cases, both patients exhibited monoclonal gammopathy. However, the two cases differ. In Case 1, a significant number of plasma cells expressing monoclonal λ light chains was found in renal tissue. We believe the monoclonal λ light chains in the blood had affected the kidney, leading to the diagnosis of MGRS. Additionally, after corticosteroid therapy, the M-protein disappeared, which may be attributed to the immunosuppressive and plasma cell secretion-inhibiting effects of corticosteroids. A previous study also reported a case of proliferative glomerulonephritis with M-protein deposits (PGNMID), a subtype of MGRS, that responded well to corticosteroid therapy (21). In contrast, in Case 2, renal biopsy showed no significant difference in light chain expression. After corticosteroid and rituximab therapy, proteinuria improved, and renal function recovered, while M-protein persisted in the blood. This indicates no correlation between M-protein in the blood and kidney damage, leading to a diagnosis of MGUS. Both MGUS and MGRS can progress to malignant plasma cell diseases or lymphoproliferative disorders. Therefore, these two patients will require close hematological follow-up in the future.

## Conclusion

The patient in Case 1 was diagnosed with IgG4-TIN combined with MGRS, which showed significant renal and hematological improvement after corticosteroid therapy. The patient in Case 2 was diagnosed with secondary membranous nephropathy combined with MGUS, and although kidney disease improved with corticosteroids and RTX treatment, M-protein persisted. Through these two clinical cases and a review of existing literature, we elucidate the spectrum of renal involvement in IgG4-related disease, underscoring the critical importance of early recognition to prevent irreversible fibrosis. In elderly patients, enhanced hematologic screening and prompt targeted interventions are essential to mitigate the risk of progression to hematologic malignancies such as multiple myeloma. These findings aim to assist clinicians in optimizing diagnostic and therapeutic strategies for this underdiagnosed disorder.

## Data Availability

The original contributions presented in the study are included in the article/supplementary material. Further inquiries can be directed to the corresponding author.

## References

[1] Simó-Perdigó M Martinez-Valle F . IgG4 related disease. Rev Esp Med Nucl Imagen Mol (Engl Ed). (2021) 40:107–14. doi: 10.1016/j.remn.2020.12.001 33547020

[2] Sánchez-Oro R Alonso-Muñoz EM Martí Romero L . Review of igG4-related disease. Gastroenterol Hepatol. (2019) 42:638–47. doi: 10.1016/j.gastrohep.2019.08.009 31722794

[3] Lu C Li S Qing P Zhang Q Ji X Tang Z . Single-cell transcriptome analysis and protein profiling reveal broad immune system activation in IgG4-related disease. JCI Insight. (2023) 8(17):e167602. doi: 10.1172/jci.insight.167602 37561593 PMC10544205

[4] Akiyama M Alshehri W Ishigaki S Saito K Kaneko Y . The immunological pathogenesis of IgG4-related disease categorized by clinical characteristics. Immunol Med. (2025) 48:11–23. doi: 10.1080/25785826.2024.2407224 39306708

[5] Ishikawa Y Terao C . Genetic analysis of IgG4-related disease. Mod Rheumatol. (2020) 30:17–23. doi: 10.1080/14397595.2019.1621000 31104539

[6] Maehara T Moriyama M Nakamura S . Pathogenesis of IgG4-related disease: a critical review. Odontology. (2019) 107:127–32. doi: 10.1007/s10266-018-0377-y 30019169

[7] Jain K Sengupta M Basu K Roychowdhury A Bandopadhyay M . IgG4 tubulointerstitial nephritis - An uncommon enemy! Indian J Pathol Microbiol. (2021) 64:556–8. doi: 10.4103/ijpm.Ijpm_687_20 34341272

[8] Boffa JJ Esteve E Buob D . Renal involvement in IgG4-related disease. Presse Med. (2020) 49:104017. doi: 10.1016/j.lpm.2020.104017 32234380

[9] Saeki T Kawano M Nagasawa T Ubara Y Taniguchi Y Yanagita M . Validation of the diagnostic criteria for IgG4-related kidney disease (IgG4-RKD) 2011, and proposal of a new 2020 version. Clin Exp Nephrol. (2021) 25:99–109. doi: 10.1007/s10157-020-01993-7 33398598 PMC7880946

[10] Buglioni A Jenkins SM Nasr SH Zhang P Gibson IW Alexander MP . Clinicopathologic features of igG4-related kidney disease. Kidney Int Rep. (2024) 9:2462–73. doi: 10.1016/j.ekir.2024.05.011 PMC11328570 39156178

[11] Gilani SI Buglioni A Cornell LD . IgG4-related kidney disease: Clinicopathologic features, differential diagnosis, and mimics. Semin Diagn Pathol. (2024) 41:88–94. doi: 10.1053/j.semdp.2023.12.001 38246802

[12] Kawano M Saeki T Ubara Y Matsui S . Recent advances in IgG4-related kidney disease. Mod Rheumatol. (2023) 33:242–51. doi: 10.1093/mr/roac065 35788361

[13] Ohta M Moriyama M Goto Y Kawano S Tanaka A Maehara T . A case of marginal zone B cell lymphoma mimicking IgG4-related dacryoadenitis and sialoadenitis. World J Surg Oncol. (2015) 13:67. doi: 10.1186/s12957-015-0459-z 25889621 PMC4350294

[14] Wu YH Wang LC Yen SH Yu WK Kao SC Kau HC . Change of serum igG4 in patients with ocular adnexal marginal zone B cell lymphoma associated with igG4-related ophthalmic disease after treatment. J Ocul Pharmacol Ther. (2017) 33:543–8. doi: 10.1089/jop.2016.0175 28514197

[15] Nishida K Sogabe Y Makihara A Senoo A Morimoto H Takeuchi M . Ocular adnexal marginal zone lymphoma arising in a patient with IgG4-related ophthalmic disease. Mod Rheumatol. (2019) 29:383–7. doi: 10.1080/14397595.2016.1216733 27686866

[16] Wang H Su T Kang L Yang L Wang S . Diffuse large B cell lymphoma in a preceding IgG4-related disease with kidney restricted lambda light chain expression: case report and literature review. BMC Nephrol. (2020) 21:315. doi: 10.1186/s12882-020-01975-7 32727411 PMC7391529

[17] Gonsalves WI Rajkumar SV . Monoclonal gammopathy of undetermined significance. Ann Intern Med. (2022) 175:Itc177–itc92. doi: 10.7326/aitc202212200 36508741

[18] Liu Y Parks AL . Diagnosis and management of monoclonal gammopathy of undetermined significance: A review. JAMA Intern Med. (2025) 185:450–6. doi: 10.1001/jamainternmed.2024.8124 PMC11975479 39960681

[19] Mouhieddine TH Weeks LD Ghobrial IM . Monoclonal gammopathy of undetermined significance. Blood. (2019) 133:2484–94. doi: 10.1182/blood.2019846782 31010848

[20] Amaador K Peeters H Minnema MC Nguyen TQ Dendooven A Vos JMI . Monoclonal gammopathy of renal significance (MGRS) histopathologic classification, diagnostic workup, and therapeutic options. Neth J Med. (2019) 77:243–54. 31582582

[21] Ogura Y Yabushita S Aihara H Tsukada H Hashiba T Furuse S . A case of proliferative glomerulonephritis with monoclonal IgG deposits (PGNMID) that responded favorably to steroid therapy. CEN Case Rep. (2022) 11:208–15. doi: 10.1007/s13730-021-00653-3 PMC9061924 34628583

